# Molecular detection and phylogenetic characterization of *Anaplasma phagocytophilum* in brown bears (*Ursus arctos*) in Slovakia

**DOI:** 10.3389/fcimb.2026.1890676

**Published:** 2026-09-09

**Authors:** Kristína Hudáková, Marián Prokeš, Monika Drážovská, Patrícia Petroušková, Katarína Franková, Lýdia Gogoľová, Alica Pavlová, Maroš Kostičák, Jakub Lipinský, Ľuboš Korytár, Ladislav Molnár, Boris Vojtek, Zuzana Hurníková, Tetiana Kuzmina, Anna Ondrejková

**Affiliations:** 1 Department of Epizootiology, Parasitology and Protection of One Health, University of Veterinary Medicine and Pharmacy in Košice, Košice, Slovakia; 2 Clinic of Birds, Exotic and Free Living Animals, University of Veterinary Medicine and Pharmacy in Košice, Košice, Slovakia; 3 Institute of Parasitology, Slovak Academy of Sciences, Košice, Slovakia; 4 I. I. Schmalhausen Institute of Zoology National Academy of Sciences of Ukraine, Kyiv, Ukraine

**Keywords:** *Anaplasma phagocytophilum*, *ankA* gene, brown bears, *groEL* gene, ticks

## Abstract

Wild animals play a crucial role in the maintenance of tick-borne pathogens, acting as reservoir and sentinel hosts within natural ecosystems. The ongoing expansion of tick populations across Europe has intensified the circulation of zoonotic tick-borne pathogens, including *Anaplasma phagocytophilum*. Although the epidemiology of this bacterium has been extensively investigated in domestic animals and small wild mammals, the contribution of large omnivores such as the brown bear (*Ursus arctos*) remains poorly understood, particularly with respect to their exposure to genetically distinct lineages with zoonotic potential, which requires further molecular characterization. This study aimed to determine the detection rate of *Anaplasma* spp. in free-ranging brown bears (n = 76) and to molecularly characterize detected *A. phagocytophilum* isolates regarding their potentially zoonotic lineages. Spleen and liver samples were screened for *Anaplasma* spp. by nested PCR targeting the *16S rRNA* gene, with subsequent molecular characterization focused on *groEL* gene and *ankA* gene. In total, 8 out of 76 samples were positive for *Anaplasma* spp., corresponding to a PCR detection rate of 10.53% (95% CI: 4.7–19.7%), with subsequent sequence analysis confirming the presence of *A. phagocytophilum*. Phylogenetic reconstruction based on *groEL* and *ankA* gene fragments allowed classification of the isolates into known European genetic lineages. This research confirms *A. phagocytophilum* exposure within the Slovak brown bear populations, identifying genetic variants relevant to public and veterinary health. These results suggest that brown bears may be exposed to *A. phagocytophilum* and could potentially play a role in its enzootic cycle, though further studies are needed.

## 1 Introduction

Wild animals play a crucial role in the ecology of tick-borne pathogens by acting as hosts for tick development and by contributing to the maintenance of enzootic transmission cycles within natural ecosystems (Kazimírová et al., 2024). The diversity, distribution, and infection status of tick populations are important determinants influencing the epidemiology of tick-borne pathogens in wildlife and domestic animals (Ayan et al., 2024). The expansion of tick populations in Europe, particularly *Ixodes ricinus*, has been associated with an increased risk of transmission of zoonotic pathogens in urban environments affecting both humans and animals (de la Fuente et al., 2008; Radzijevskaja et al., 2026).

*Anaplasma phagocytophilum* is an obligate intracellular, multi-host pathogen transmitted primarily by *I. ricinus* ticks. It causes human granulocytic anaplasmosis, tick-borne fever in ruminants and granulocytic anaplasmosis in domestic and wild animals and is considered an emerging zoonotic pathogen across Europe (Stuen et al., 2013; Matei et al., 2019). The bacterium persists in complex enzootic cycles involving multiple vertebrate hosts, although the relative contribution of different wildlife species to pathogen maintenance remains incompletely understood (Tsao et al., 2021).

In Europe, *A. phagocytophilum* has been detected in a wide range of wildlife species, and molecular evidence indicates that cervids and small mammals are important reservoir hosts (Stuen et al., 2013; Rosso et al., 2017; Matei et al., 2018). However, increasing evidence suggests that additional wildlife species may also be exposed and potentially involved in transmission cycles, including large carnivores and omnivores (Dugat et al., 2015).

The brown bear (*Ursus arctos*) represents an important component of the Carpathian ecosystem. Following historical population decline, the species has recovered and now forms part of a continuous transboundary population across the Carpathians (Rigg and Adamec, 2007; Paule et al., 2015; Fedorca et al., 2019). Due to its large home range, long lifespan, and opportunistic feeding behavior, the brown bear is frequently exposed to tick-infested environments and may represent a useful sentinel species for monitoring tick-borne pathogens (Matei et al., 2021; McLellan et al., 2021).

Although *A. phagocytophilum* has been well studied in Central Europe in domestic animals, including dogs and cattle, and in cervids, its occurrence and genetic diversity in brown bears remain poorly characterized (Sainz et al., 2015; Kazimírová et al., 2018; Hoffman et al., 2025). The first molecular detection of *A. phagocytophilum* in Slovak brown bears was reported by Víchová et al. (2010), but information on strain diversity and phylogenetic structure in this host species is still limited.

Molecular identification of *Anaplasma* spp. commonly relies on the *16S rRNA* gene (Dumler et al., 2001); however, this marker provides limited resolution for strain differentiation (Caudill and Brayton, 2022). Therefore, more variable genetic loci such as *groEL* and *ankA* are increasingly used to resolve phylogenetic relationships and ecotype structure, and to assess potential zoonotic lineages (Scharf et al., 2011; Jahfari et al., 2014). Previous studies have demonstrated that *A. phagocytophilum* exhibits marked genetic diversity and host-associated structuring, suggesting the existence of distinct ecological cycles with variable zoonotic potential (Fröhlich et al., 2023).

Therefore, this study aimed to determine the PCR detection rate of *Anaplasma* spp. in free-ranging brown bears in Slovakia and molecularly characterize obtained *A. phagocytophilum* strains using selected genetic markers (*groEL* and *ankA*) in order to assess strain diversity, zoonotic potential and their phylogenetic relationships within known European lineages.

## 2 Materials and methods

### Sample collection

**Figure 1:**
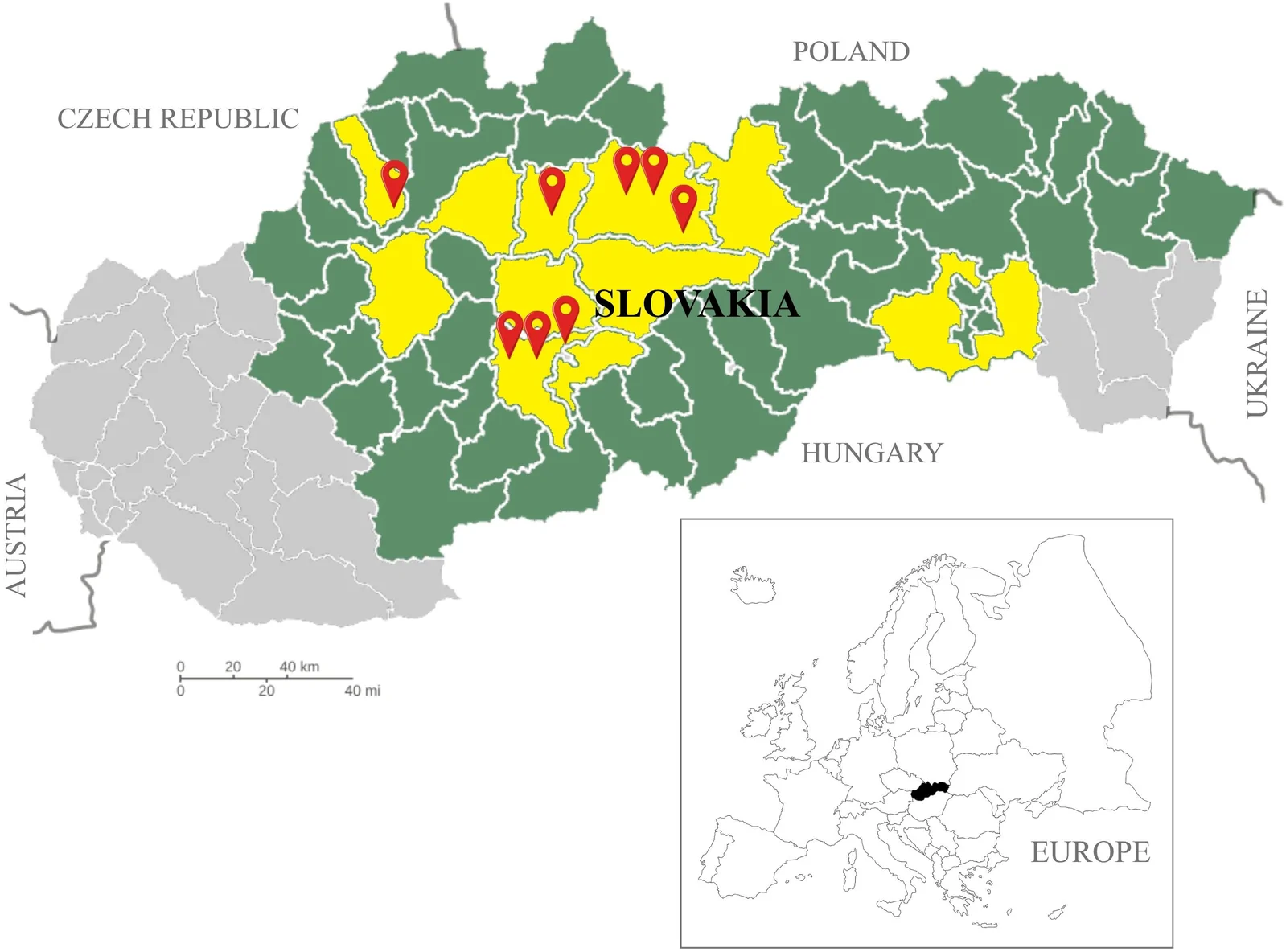
Geographical origin of samples. Districts of Slovakia: with legally authorised bear population management measures (green and yellow); without authorised bear population management measures (grey); study sampling districts (yellow). The red point symbol represents localities with positive samples.

Spleen and liver tissue samples from 76 brown bears (*Ursus arctos*) were collected from April to October 2025, during legally authorized population management measures conducted in Slovakia. Demographic data are shown in **Supplementary Table 1**. Samples came from 11 different Slovakia district (**Figure 1**). In total, 72 spleen samples and 4 liver samples were included in the study. The population control measures were implemented following the declaration of an emergency situation related to the occurrence of brown bears in selected districts of Slovakia, as approved by the Government of the Slovak Republic (Government Resolution No. 182/2025).

### Molecular detection and sequencing

Genomic DNA was extracted from tissue samples using the DNeasy Blood & Tissue Kit (Qiagen, Hilden, Germany) according to the manufacturer’s protocol. Approximately 10 mg of spleen tissue and 25 mg of liver tissue were used for DNA isolation. DNA was eluted in a final volume of 100 µL elution buffer and stored at 4°C until molecular analysis. DNA concentration and purity were assessed using spectrophotometric analysis with a NanoDrop One instrument (Thermo Fisher Scientific, Waltham, MA, USA). To verify the quality of the extracted DNA and to rule out the presence of PCR inhibitors, all 76 samples were subjected to an internal control PCR targeting the host mitochondrial *cytochrome b* gene (486bp fragment). Amplification was performed using the universal mammalian primers L14724 (5′-CGAAGCTTGATATGAAAAACCATCGTTG-3′) and H15149 (5′- AAACTGCAGCCCCTCAGAATGATATTTGTCCTCA-3′) according to Irwin et al. (1991). Successful amplification of the target fragment was obtained for all 76 samples, confirming high-quality DNA templates throughout the study. A representative agarose gel showing 17 randomly selected samples is shown in **Supplementary Figure 1**.

Screening for *Anaplasma* spp. was performed by nested PCR targeting the *16S rRNA* gene. Positive samples were further characterized by *groEL* and *ankA* gene analyses to determine their ecotype and cluster affiliation. All PCR reactions were performed in a total reaction volume of 25 µL containing 12.5 µL of 2× PPP Master Mix (Top-Bio, Vestec, Czech Republic), 1 µL of each primer (30 µM), 9.5 µL of PCR molecular-free water, and 1 µL of DNA template. Nested PCR was used for amplification of all partial gene fragments. Specific primers and cycling condition are described in **Table 1**.

**Table 1:** Specific primers and cycling condition for amplification *16S rRNA*, *groEL* and *ankA* gene fragments.

Gene	Primers 5′-3′	Cycle conditions*	References
*16S rRNA*	F1:CACATGCAAGTCGAACGGATTATTC R1:TTCCGTTAAGAAGGATCTAATCTCC F2:AACGGATTATTCTTTATAGCTTGCT R2:GGCAGTATTAAAAGCAGCTCCAGG	initial denaturation: 94°C 2 min 40 cycles (nested reaction: 30 cycles): 94°C 30 sec, 55°C 30 sec, 72°C 1 min Product size (1^st^/2^nd^ reaction): 932bp/546bp	Silaghi et al., 2011
*groEL*	F:ATGGTATGCAGTTTGATCGC R1:TCTACTCTGTCTTTGCGTTC R2:TTGAGTACAGCAACACCACCGGAA	initial denaturation: 95°C 5 min 40 cycles: 95°C 30 sec, 55°C 30 sec, 72°C 45 sec Product size (1^st^/2^nd^ reaction): 625bp/574bp	Alberti et al., 2005
*ankA*	F1:TRSTGTAAATGAAGAAATTACAACTYC R1:GMCTTTAGTAGTAYTYTACATG F2:TYTGACCGCTGAAGCACTAA R2: GMAGCMAGATGCAGTAACGW	initial denaturation: 95°C 3 min 34 cycles 95°C 10 sec, 50°C 10 sec (nested reaction: 58°C 20 sec), 72°C 45 sec Product size (1^st^/2^nd^ reaction): 774bp/687bp	this study

^*^ All PCR reactions: final extension at 72 °C 5 min.

*A. phagocytophilum* DNA previously obtained from sheep in our laboratory and confirmed by Sanger sequencing (unpublished data) served as positive control in all PCR assay; no-template control was used as negative control. PCR products were visualized by electrophoresis on a 1.0% agarose gel stained with GelRed Nucleic Acid Stain (Biotium, Fremont, CA, USA) (**Supplementary Figures 2**-**4**). Positive PCR products were purified and subjected to Sanger sequencing by Microsynth (Balgach, Switzerland). Nucleotide sequence analysis was performed using Geneious Prime v2025.1.3 (Biomatters Ltd., Auckland, New Zealand). Obtained sequences were compared with reference sequences available in the GenBank database using the BLASTn (https://blast.ncbi.nlm.nih.gov/Blast.cgi; accessed on 7 May 2026) and submitted to the GenBank database using the NCBI submission portal (https://www.ncbi.nlm.nih.gov/WebSub/; accessed on 15 May 2026) under accession numbers PZ415575–PZ415587.

### Phylogenetic analysis

Phylogenetic analyses were performed using reference sequences retrieved from the GenBank database together with sequences obtained in this study, separately for *groEL* partial gene fragment (568bp) and *ankA* partial gene fragment (687bp). Multiple-sequence alignments were generated using the ClustalW algorithm implemented in MEGA software version 12.1. Phylogenetic trees were constructed using the maximum likelihood method and Tamura-3 (T92: for *groEL* gene fragment) and Kimura-2 (K2: for *ankA* gene fragment) with the proportion of invariant sites (I). The robustness and statistical support of the tree topologies were evaluated by bootstrap analysis with 1000 replications.

## 3 Results

Out of 76 tissue samples obtained from brown bears, including 72 spleen samples and 4 liver samples, PCR amplification targeting the *16S rRNA* gene detected *Anaplasma* spp. DNA in 8 samples, corresponding to a PCR detection rate of 10.53% (95% CI: 4.7–19.7%), including seven spleen samples and one liver sample. Regarding the demographic profile of the positive animals, they comprised 4 males and 4 females, with ages ranging from 1 to 8 years. Identified nucleotide sequences are 100% identical to each other and to *16S rRNA* gene sequence of *A. phagocytophilum* isolated from various wild and domestic animals (KX180948 *Vulpes vulpes*, AY527213 *Equus caballus*, *PZ231809 Capra aegagrus hircus*, MT860417 *Canis lupus*, EU839848 *Capreolus capreolus*, PZ231799 *Ovis aries*, MT860416 *Canis aureus*, MF288942 *Felis catus* and in *Ixodes ricinus* HQ629917).

**Figure 2:**
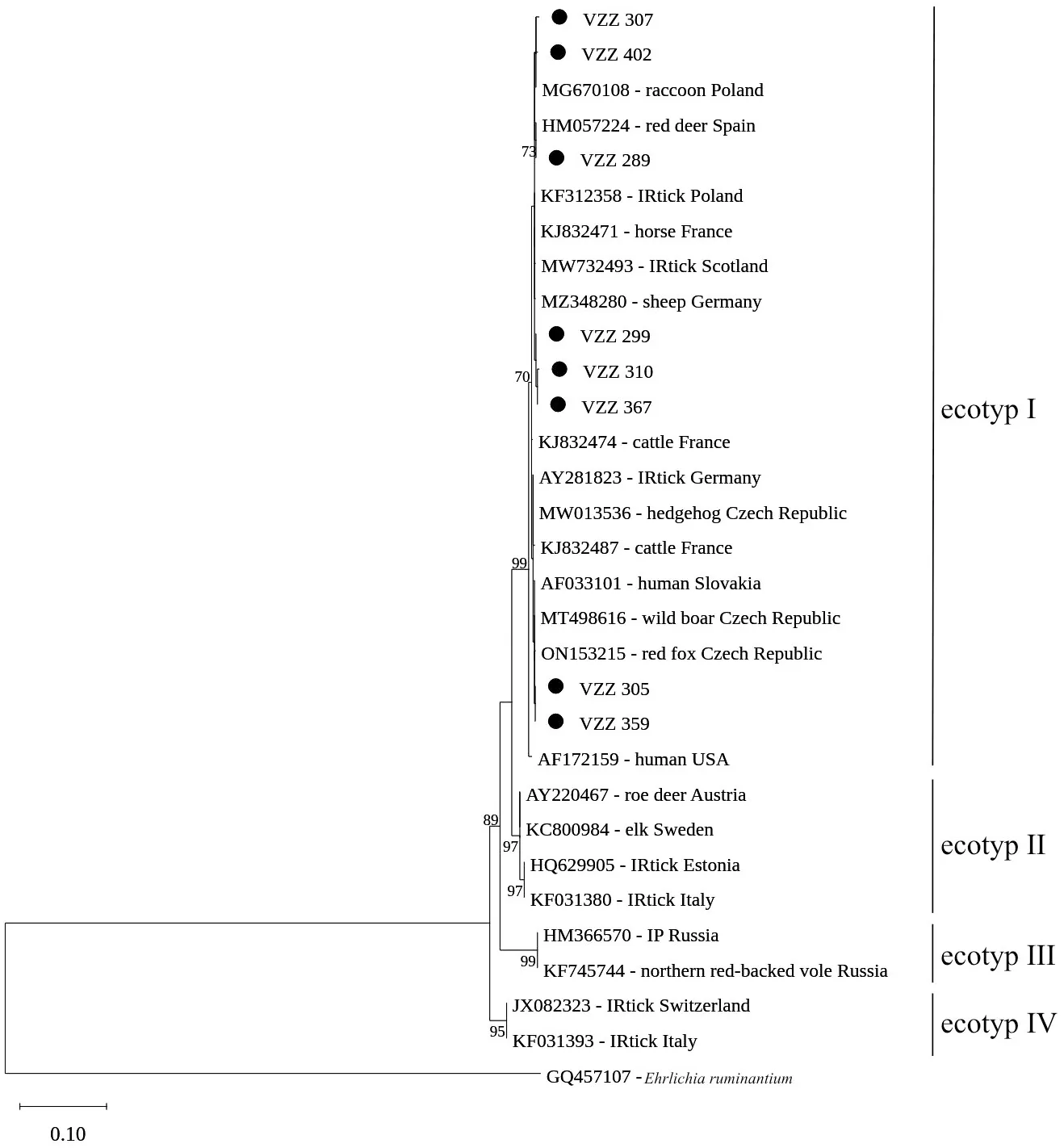
Phylogenetic tree of *groEL* partial gene (568bp) of *Anaplasma phagocytophilum*, constructed by MEGA v12.1 using the Maximum Likelihood method and the Tamura-3 parameter model (T92) with the proportion of invariant sites (I). Statistical support was provided by bootstrapping with 1000 replicates; bootstrap values higher than 70 are shown at the branch point. Sequences obtained in this study are highlighted by black dots. Ecotypes I-IV are shown on the right side of the phylogenetic tree.

Following amplification of the partial *groEL* gene was successful in all eight positive samples. Comparative analysis of the obtained sequences revealed nine single nucleotide polymorphisms that did not result in amino acid changes (**Supplementary Table 2**). Phylogenetic analysis clustered all obtained sequences with previously described ecotype I isolates of *A. phagocytophilum* (**Figure 2**).

**Figure 3:**
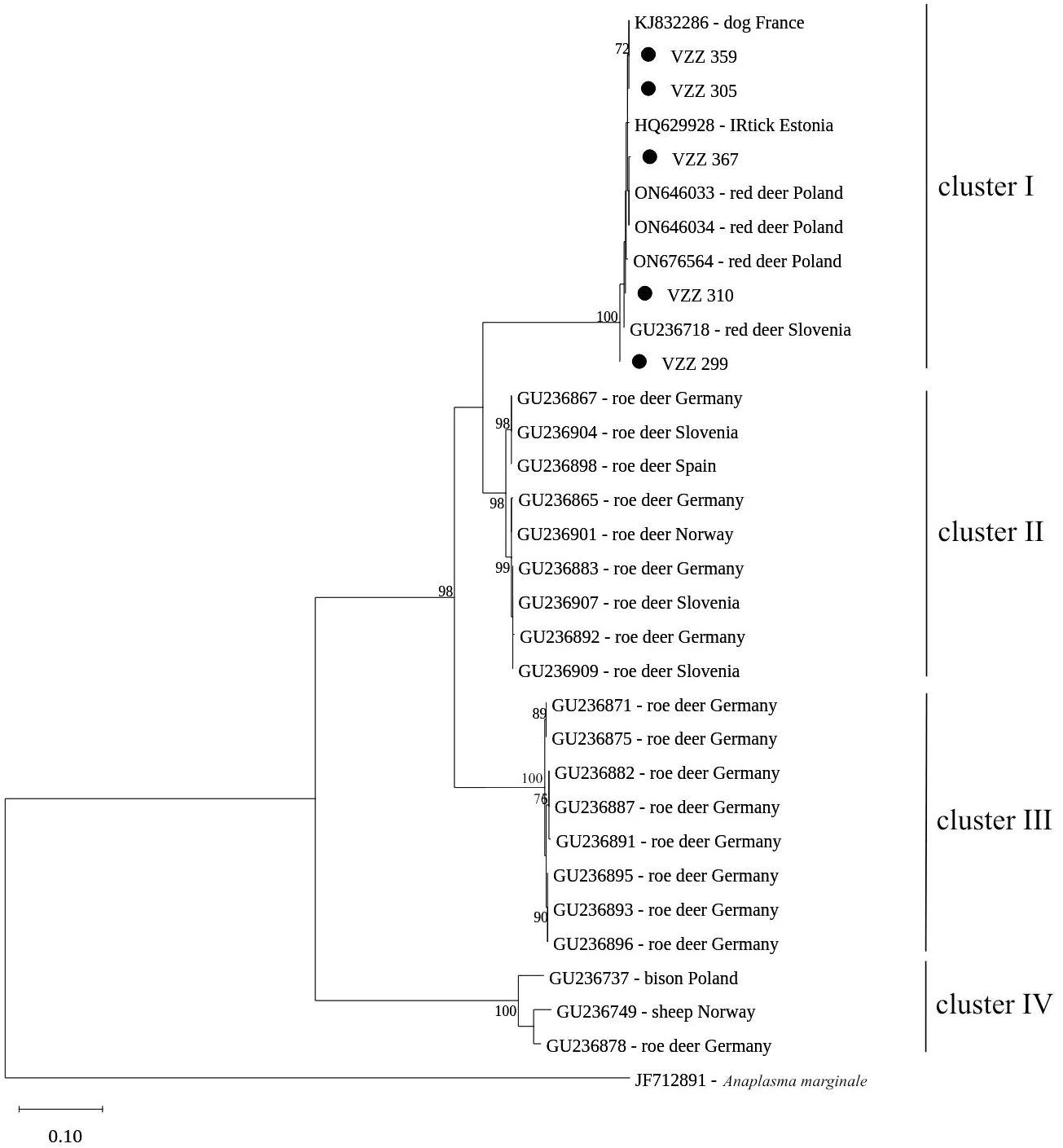
Phylogenetic tree based on the *ankA* partial gene fragments of *Anaplasma phagocytophilum* (687bp) was constructed by MEGA v12.1 using the Maximum Likelihood method and the Kimura-2 parameter model with the proportion of invariant sites (K2+I). Bootstrap analysis values ​​(1000 replications) higher than 70 are shown at the branch point. Sequences obtained in this study are highlighted by black dots. Clusters I-IV are shown on the right side of the phylogenetic tree.

The partial fragment of *ankA* gene was successfully amplified in five samples. Multiple sequence alignment analysis confirmed 4 distinct strains containing six single-nucleotide polymorphisms, five of which led to amino acid substitutions (**Supplementary Table 3**). After phylogenetic analysis, all obtained sequences were classified into *A. phagocytophilum ankA* cluster I (**Figure 3**) and showed high sequence similarity (99.13-100%) to previously published sequences detected in carnivores (GU236819, GU236837) as well as in humans (GU236801).

## 4 Discussion

Free-ranging European brown bears (*Ursus arctos*) are exposed to multiple infectious agents circulating in sylvatic ecosystems, including vector-borne pathogens (Vitásková et al., 2019). Due to their large body size and frequent exposure to ticks, brown bears may serve as useful sentinel hosts for monitoring tick-borne pathogens (Matei et al., 2021).

Molecular detection of *A. phagocytophilum* in European brown bears was first reported in Slovakia by Víchová et al. (2010), who detected the pathogen in tissue samples using *16S rRNA* gene analysis. Subsequent studies confirmed the occurrence of *A. phagocytophilum* in brown bears and other European wild carnivores, suggesting their potential role as incidental or sentinel hosts (André, 2018; Matei et al., 2021).

In the present study, *A. phagocytophilum* DNA was detected in 8 out of 76 examined brown bears. Based on BLAST and phylogenetic analyses of the *groEL* gene sequences, all sequences obtained in this study showed the highest similarity to *A. phagocytophilum* strains previously reported from Europe and deposited in the GenBank database, supporting their classification within the European ecotype I lineage (Jahfari et al., 2014). Similarly, *ankA* gene sequences obtained in this study clustered within the cluster I lineage, consistent with a zoonotic strain profile circulating in Europe (Scharf et al., 2011). These findings indicate phylogenetic affiliation with strains commonly associated with zoonotic transmission cycles and suggest that brown bears in Central Europe may be exposed to *A. phagocytophilum* lineages also detected in ticks, wildlife, domestic animals and humans. Although the reservoir competence of brown bears remains unclear, the detection of ecotype I strains supports their potential role as sentinel hosts for assessing exposure to *A. phagocytophilum*.

Moustafa et al. (2015) detected *Anaplasma* DNA in 2 of 13 wild brown bears in Japan based on *16S rRNA* analysis. However, the absence of additional genetic markers limited further characterization of this lineage. In contrast, the present study provides multilocus characterization using *groEL* and *ankA* genes, enabling a more detailed assessment of *A. phagocytophilum* genetic affiliation. Recent studies have shown that analysis of the *groEL* and *ankA* genes provides more detailed characterization of *A. phagocytophilum* strains compared with the conserved *16S rRNA* gene, allowing assignment to specific ecotypes and *ankA* clusters associated with host adaptation and zoonotic relevance (Scharf et al., 2011; Víchová et al., 2014; Jaarsma et al., 2019). Ecotype I and *ankA* cluster I lineages have repeatedly been reported in humans, domestic animals and several wild carnivores across Europe, whereas other lineages appear to be more frequently associated with wildlife hosts (Lesiczka et al., 2023; Myczka et al., 2024; Pavlak et al., 2025). Although *A. phagocytophilum* is a recognized pathogen of humans and several domestic and wild mammals, clinical anaplasmosis has not been documented in brown bears. Therefore, the biological significance of *A. phagocytophilum* detection in this species remains unclear, and brown bears should currently be regarded as potential incidental or sentinel hosts rather than confirmed reservoir hosts.

Previous serological studies in Scandinavian brown bears further support frequent exposure of this species to tick-borne pathogens and their potential value as sentinel animals (Paillard et al., 2015). Tick abundance and distribution are influenced by ecological conditions, host availability, and management practices, which may affect the risk of tick-borne pathogen transmission and highlight the importance of integrated tick surveillance and control strategies (Ahmad et al., 2025). These findings indicate frequent exposure of bears to tick-borne pathogens and support the potential usefulness of large carnivores as indicators of ecological changes affecting tick distribution and pathogen circulation. Together, these studies suggest that brown bears are regularly exposed to infected ticks and may serve as incidental or sentinel hosts for monitoring tick-borne pathogen circulation.

This study has several limitations that should be considered when interpreting the results. First, the sample size of 76 brown bears, of which 8 tested positive for *A. phagocytophilum*, was relatively limited, and samples were obtained opportunistically from legally hunted individuals rather than through a structured epidemiological survey and therefore the observed PCR detection rate may not fully represent the occurrence of infection in the wider brown bear population. Future studies based on larger sample sizes and systematic sampling designs, including defined geographic and demographic categories, would provide a more representative assessment of pathogen occurrence and associated risk factors. Second, the nested PCR approach used in this study was qualitative and did not allow quantification of bacterial DNA. Furthermore, this method does not allow discrimination between samples containing different amounts of bacterial DNA or between active infection and the presence of residual bacterial DNA. Future application of quantitative PCR methods could provide additional information on bacterial load and help clarify the biological significance of positive detections. Third limitation of this study is the absence of a dedicated extraction negative control. However, it is worth noting that in each processed extraction batch, the majority of samples consistently yielded negative results in the nested PCR, with only one or two samples testing positive. This low positivity rate within individual extraction runs suggests that widespread cross-contamination during the isolation phase was unlikely, further supported by the consistently negative no-template controls in all subsequent PCR runs. Fourth, histopathological examination of tissue samples was not performed; therefore, macroscopic or microscopic tissue changes potentially associated with *A. phagocytophilum* detection could not be evaluated. Consequently, it remains unclear whether the detected bacterial DNA was associated with active tissue infection or pathological changes. Future investigations integrating molecular analyses with histopathology and complementary immunological approaches could provide a more comprehensive understanding of host–pathogen interactions in brown bears.

Despite these limitations, the use of multiple genetic markers enabled the characterization of *A. phagocytophilum* strains detected in brown bears and provides novel data on its genetic diversity, addressing an important knowledge gap in this understudied wildlife host.

## 5 Conclusion

The detection of *A. phagocytophilum groEL* sequences clustering within ecotype I and *ankA* cluster I variants in brown bears indicates exposure of this species to lineages previously associated with zoonotic relevance in Europe. However, the present findings do not allow conclusions regarding the reservoir competence or contribution of brown bears to the maintenance of *A. phagocytophilum* transmission cycles.

Wildlife reservoirs are considered essential for the long-term maintenance of *A. phagocytophilum* in nature, with cervids and small mammals recognized as key hosts, while the epidemiological role of large carnivores remains poorly understood. Due to their exposure to ticks and their use of diverse habitats, brown bears may represent valuable sentinel hosts for monitoring the circulation of tick-borne pathogens.

Increasing overlap between brown bear habitats and human environments in recent years may increase opportunities for human exposure to tick-infested environments. However, further studies are needed to determine whether brown bears have any epidemiological role beyond incidental exposure and to better understand their significance within the ecology of *A. phagocytophilum* in Central Europe.

## Data Availability

The dataset presented in this study can be found in an online repository (https://www.ncbi.nlm.nih.gov/genbank/) under the accession number PZ415575-PZ415587. Further inquiries can be directed at the corresponding author.
