# Supplementary material for: Molecular detection and phylogenetic characterization of Anaplasma phagocytophilum in brown bears (Ursus arctos) in Slovakia
Source: Front Cell Infect Microbiol. 2026 Sep 9;16:1890676. doi: 10.3389/fcimb.2026.1890676 (PMC13597328; doi:10.3389/fcimb.2026.1890676)
Supplement: Supplementary file 1 [file DataSheet1.docx]

Supplementary Material

# 1 Supplementary Data

The dataset presented in this study can be found in an online repository (<https://www.ncbi.nlm.nih.gov/genbank/>) under the accession number PZ415575- PZ415587. Further inquiries can be directed at the corresponding author.

# 2 Supplementary Figures and Tables

## 2.1 Supplementary Figures

**Supplementary Figure 1.** PCR amplification of the host internal control (*cyt-b*). Representative agarose gel showing the PCR products of the *cytochrome b* gene used to verify DNA quality and the absence of PCR inhibitors. Lane L: DNA 100bp ladder; Lane NC: negative control (tick DNA); Lane PC: positive control (horse DNA); Lanes 1–17: randomly selected representative bear samples.

**Supplementary Figure 2.** PCR amplification of the *16S rRNA* gene. Lane L: DNA 100bp ladder; Lane NC: negative control; Lane PC: positive control; Lanes 1–9: randomly selected representative negative samples; Lanes 10–17: positive samples.

**Supplementary Figure 3.** PCR amplification of the *groEL* gene. Lane L: DNA 100bp ladder; Lane NC: negative control; Lane PC: positive control; Lanes 1–8: positive samples; Lanes 9–17: randomly selected representative negative samples.

**Supplementary Figure 4.** PCR amplification of the *ankA* gene. Lane L: DNA 100bp ladder; Lane NC: negative control; Lane PC: positive control; Lanes 1–5: positive samples; Lanes 6–17: randomly selected representative negative samples.

## 2.3 Supplementary Tables

| Sample ID |  | Demographic data | | Sample ID |  | Demographic data | |
| --- | --- | --- | --- | --- | --- | --- | --- |
|  |  | sex | age (years) |  |  | sex | age (years) |
| VZZ 235 |  | male | NA | VZZ 360 |  | NA | NA |
| VZZ 283 |  | female | NA | VZZ 361 |  | female | 3 |
| VZZ 284 |  | female | 6 | VZZ 362 |  | male | 9 |
| VZZ 285 |  | male | 2 | VZZ 363 |  | female | 2 |
| VZZ 286 |  | NA | NA | VZZ 364 |  | female | 1 |
| VZZ 287 |  | male | 2 | VZZ 365 |  | male | 2 |
| VZZ 288 |  | NA | NA | VZZ 366 |  | female | 4 |
| VZZ 289 |  | male | 4 | VZZ 367 |  | female | 4 |
| VZZ 296 |  | NA | 5 | VZZ 368 |  | male | 8 |
| VZZ 297 |  | NA | 3 | VZZ 369 |  | female | 3 |
| VZZ 298 |  | male | 5 | VZZ 370 |  | NA | 4 |
| VZZ 299 |  | male | 3 | VZZ 371 |  | male | 8 |
| VZZ 300 |  | NA | 20 | VZZ 372 |  | male | 10 |
| VZZ 301 |  | male | 2 | VZZ 373 |  | female | 5 |
| VZZ 302 |  | female | 2 | VZZ 374 |  | female | 3 |
| VZZ 303 |  | male | 2 | VZZ 375 |  | female | 6 |
| VZZ 304 |  | male | 2 | VZZ 376 |  | male | 12 |
| VZZ 305 |  | male | 2 | VZZ 392 |  | female | 6 |
| VZZ 306 |  | male | 8 | VZZ 393 |  | male | 10 |
| VZZ 307 |  | female | 8 | VZZ 394 |  | male | 6 |
| VZZ 308 |  | male | 3 | VZZ 395 |  | NA | NA |
| VZZ 309 |  | female | 6 | VZZ 396 |  | male | 4 |
| VZZ 310 |  | female | 1 | VZZ 397 |  | female | NA |
| VZZ 311 |  | female | 10 | VZZ 398 |  | female | NA |
| VZZ 312 |  | male | 6 | VZZ 399 |  | NA | NA |
| VZZ 313 |  | male | 1 | VZZ 400 |  | male | 4 |
| VZZ 314 |  | female | 1 | VZZ 401 |  | male | NA |
| VZZ 315 |  | female | 6 | VZZ 402 |  | female | 8 |
| VZZ 316 |  | NA | NA | VZZ 403 |  | female | 10 |
| VZZ 317 |  | female | 3 | VZZ 404 |  | male | NA |
| VZZ 318 |  | female | 17 | VZZ 405 |  | NA | NA |
| VZZ 319 |  | female | 5 | VZZ 406 |  | male | 3 |
| VZZ 320 |  | female | 4 | VZZ 418 |  | female | 25 |
| VZZ 355 |  | female | 2 | VZZ 419 |  | male | 1 |
| VZZ 356 |  | female | 16 | VZZ 420 |  | female | 1 |
| VZZ 357 |  | male | 15 | VZZ 425 |  | male | 3 |
| VZZ 358 |  | female | 9 | VZZ 457 |  | NA | NA |
| VZZ 359 |  | male | 3 | VZZ 458 |  | NA | NA |

Supplementary Table 1. Demographic data of tested brown bear population. NA – data not available

| Sample ID | GenBank accession number | *groEL* amino acid / nucleotide position | | | | | | | | |
| --- | --- | --- | --- | --- | --- | --- | --- | --- | --- | --- |
|  |  | 30/90-92 | 54/162-164 | 68/204-206 | 70/210-212 | 85/255-257 | 90/270-272 | 121/363-365 | 156/468-470 | 181/543-545 |
| VZZ 289 | PZ415575 | Phe / TTC | Arg / AGA | Leu / CTG | Thr / ACA | Val / GTA | Phe / TTT | Asp / GAT | Arg / AGG | Ala / GCG |
| VZZ 299 | PZ415576 | Phe / TTC | Arg / AGA | Leu / CTG | Thr / ACG | Val / GTG | Phe / TTT | Asp / GAT | Arg / AGG | Ala / GCG |
| VZZ 305 | PZ415577 | Phe / TTT | Arg / AGA | Leu / CTG | Thr / ACG | Val / GTA | Phe / TTC | Asp / GAC | Arg / AGG | Ala / GCA |
| VZZ 307 | PZ415578 | Phe / TTC | Arg / AGG | Leu / CTT | Thr / ACG | Val / GTA | Phe / TTT | Asp / GAT | Arg / AGA | Ala / GCG |
| VZZ 310 | PZ415579 | Phe / TTC | Arg / AGA | Leu / CTG | Thr / ACG | Val / GTG | Phe / TTT | Asp / GAC | Arg / AGG | Ala / GCA |
| VZZ 359 | PZ415580 | Phe / TTT | Arg / AGA | Leu / CTG | Thr / ACG | Val / GTA | Phe / TTC | Asp / GAC | Arg / AGG | Ala / GCA |
| VZZ 367 | PZ415581 | Phe / TTC | Arg / AGA | Leu / CTG | Thr / ACG | Val / GTG | Phe / TTT | Asp / GAT | Arg / AGG | Ala / GCA |
| VZZ 402 | PZ415582 | Phe / TTC | Arg / AGA | Leu / CTT | Thr / ACG | Val / GTA | Phe / TTT | Asp / GAT | Arg / AGG | Ala / GCA |

Supplementary Table 2. Differences in nucleotide and amino acid profile detected in obtained partial (574bp) *groEL* gene sequences from brown bears.

| Sample ID | GenBank accession number | *ankA* amino acid / nucleotide position | | | | | |
| --- | --- | --- | --- | --- | --- | --- | --- |
|  |  | 13 / 38-40 | 16 / 47-49 | 64 / 191-193 | 83/ 248-250 | 158 / 473-475 | 168 / 503-505 |
| VZZ 299 | PZ415583 | Ala / GCA | Ala / GCG | Thr / ACC | Leu / CTT | Leu / TTA | Gln / CAG |
| VZZ 305 | PZ415584 | Val / GTA | Thr / ACG | Pro / CCC | Pro / CCT | Leu / TTG | Arg / CGG |
| VZZ 310 | PZ415585 | Ala / GCA | Thr / ACG | Thr / ACC | Pro / CCT | Leu / TTG | Arg / CGG |
| VZZ 359 | PZ415586 | Val / GTA | Thr / ACG | Pro / CCC | Pro / CCT | Leu / TTG | Arg / CGG |
| VZZ 367 | PZ415587 | Ala / GCA | Thr / ACG | Pro / CCC | Leu / CTT | Leu / TTG | Gln / CAG |

Supplementary Table 3. Differences in nucleotide and amino acid profile detected in obtained partial (687bp) *ankA* gene sequences from brown bears.
